# Complexity of Clathrin Coat Dynamics in Endocytosis

**DOI:** 10.1111/tra.70052

**Published:** 2026-10-24

**Authors:** Anne‐Laure Boinet, Aurélien Roux, Marko Kaksonen

**Affiliations:** ^1^ Department of Biochemistry University of Geneva Geneva Switzerland

## Abstract

Clathrin‐mediated endocytosis is a process by which eukaryotic cells internalise a wide range of molecules such as signalling factors, nutrients and membrane proteins, via the formation of a plasma‐membrane‐derived vesicle. This complex mechanism involves a coordinated assembly of the scaffold protein clathrin and more than 50 other endocytic proteins at endocytic sites. Clathrin polymerises into sturdy supramolecular lattices, yet its assembly is highly dynamic and controlled by the DnaJ/Hsc70 uncoating machinery. Although clathrin turnover at endocytic sites was proposed early on to allow membrane deformation to occur, more recent studies have started hinting at possible regulatory functions. In this review, we summarise and discuss the key findings that have advanced our understanding of the role played by clathrin dynamics, hoping to shed light on the complex regulation of the endocytic process.

## Graphical abstract

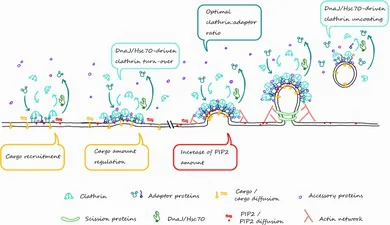

## 1 Introduction

Clathrin‐mediated endocytosis is a major pathway eukaryotic cells use for the selective uptake of a wide range of molecules, from hormones and transmembrane proteins to lipids and nutrients. It is a highly conserved internalisation route found across the eukaryotic phylogenetic tree, making it one of the most studied trafficking pathways across model organisms from mammals to plants and fungi, which has provided a good understanding of the inner workings of this complex process.

Clathrin‐mediated endocytosis involves the controlled deformation of the plasma membrane into a cargo‐filled vesicle and is characterised by the sequential and tightly regulated assembly of over 50 proteins into a complex structure. These proteins coordinate their activities to (1) initiate the endocytic process at specific locations on the plasma membrane; (2) select and capture molecules to be internalised; (3) deform the plasma membrane in an inward orientation; and (4) cut the newly formed clathrin‐coated vesicle from the plasma membrane, without compromising its integrity (Figure 1). After scission, the endocytic vesicles are rapidly uncoated and are subsequently transported to fuse with endosomes. In 1964, Roth and Porter [1], who used electron microscopy imaging to track the internalisation of electron‐dense yolk granules in mosquito oocytes, described for the first time the characteristic “bristle coats” decorating the endocytic pits and vesicles. The main component of the bristle coats, clathrin, was later identified by Barbara Pearse [2]. Subsequent work, in parallel with progress in imaging techniques, has gradually completed the picture first drawn by these founding studies, progressively identifying and characterising the many pieces of the endocytic puzzle, from adaptor proteins to the myriad of effectors and regulatory proteins, as well as the specific lipid species required for coat formation. Although the structural properties and sequential recruitment of the key players of the endocytic machinery have been described in great detail over the years, our understanding of the many parameters controlling their assembly remains poor. This is also true for clathrin, despite it being arguably one of the best characterised proteins of membrane traffic in general. While clathrin's assembly at endocytic sites is well understood from a structural standpoint, how this assembly is controlled dynamically as the endocytic vesicle is formed is still unclear. Although it was long thought to have a structural support function for the forming vesicle, a growing body of evidence is highlighting a more dynamic role for clathrin in regulating the progression of the endocytic event. In this review, we focus on the dynamic properties of clathrin assembly at endocytic sites and discuss how these properties may govern endocytic progression. We summarise experimental data pointing at potential roles of clathrin exchange in membrane deformation, cargo recruitment as well as endocytic site architectural organisation, and discuss how these various functions might occur concomitantly to ensure successful endocytic vesicle formation.

**FIGURE 1:**
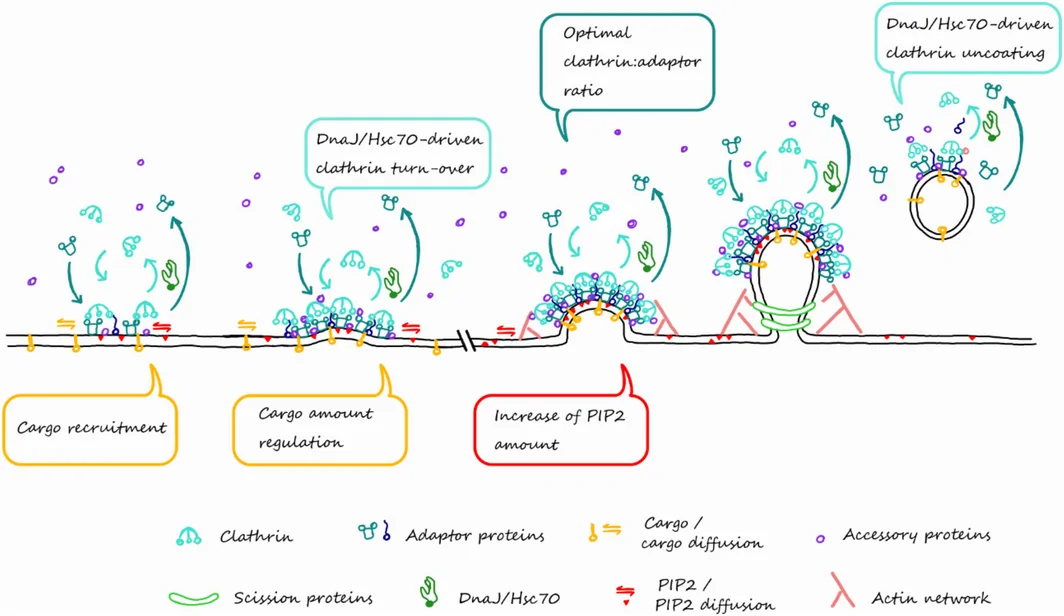
Clathrin dynamics during endocytosis. Dynamic exchange of clathrin triskelia and other endocytic proteins may be critical for several processes taking place during the maturation of endocytic vesicles.

## 2 Assembly and Disassembly of Clathrin Coats During Endocytosis

### 2.1 Structure of Clathrin and Clathrin Lattices

The conservation of clathrin is exemplified by its highly recognisable triskelion shape in many organisms. Clathrin triskelion is a heteromeric complex, composed of three clathrin heavy chains and three clathrin light chains. Clathrin assembles spontaneously into triskelia that constitute the smallest clathrin assemblies that can be found in cells [3, 4, 5].

The heavy chain structure is composed of five regions: first, a globular and partially disordered domain at the N‐terminus referred to as terminal domain, followed by three alpha‐helical segments referred to as the ankle, distal segment and proximal segment. These are followed by a trimerization domain and a short unstructured tail at the carboxyl end. The conformation of these segments gives rise to a triskelion‐shaped complex, in which all terminal domains form the end of the three ‘legs’ bending towards the concave side [6]. While sturdy overall, these legs still have some flexibility. Although the vertices and proximal segment do not bend at all, the knees and distal segment can adopt slightly different bending angles, which propagate into a wide displacement of the terminal domains [7]. These properties allow for clathrin self‐assemble into polygonal lattices, characterised by varying curvatures and sizes. This ultrastructural assembly was shown to depend heavily on distal‐proximal leg interactions [8]. The curvature is dictated by the numbers of pentagonal openings in the otherwise hexagonal array, the two extremes being 0, leading to the formation of a fully hexagonal flat lattice, and 12, corresponding to clathrin's assembly into a fully closed cage. The number of hexagons in the structure, on the other hand, dictates its overall size. These structural properties entail that a change in clathrin coat curvature over time (i.e., from a flat lattice to a highly curved coated pit) can only happen via an increase in the number of pentagons contained in the structure, which depends on a large‐scale reorganisation of the individual clathrin triskelia within the coat. In other words, to accommodate for an increase in membrane curvature, the clathrin coat should present some dynamicity in its subunit assembly. However, the exact interplay between clathrin assembly dynamics and membrane deformation during endocytosis is still unclear, and has led to opposing models, each supported by seemingly contradictory results. These contradictions stem in part from the challenge of replicating in vitro the level of regulation and complexity observed for clathrin assemblies in cells. It is also unclear how much of these contradictions are coming from interpretations of results through opposing models, and whether it could be solved by more accurate models, compatible with all data.

### 2.2 Clathrin Cage Uncoating Mechanism

The uncoating of the nascent endocytic vesicles is crucial for their subsequent fusion with their destination compartment [9]. However, clathrin baskets are characterised by a high stability in vitro. They were shown to remain intact in buffer for extended periods of time, and across a wide range of temperatures, pH values and concentrations, when assembled in presence of clathrin adaptors [10, 11]. Therefore, disassembling clathrin cages requires the ATP‐dependent activity of an uncoating machinery. In 1982, Patzer et al. showed that the disassembly of clathrin baskets required incubation with ATP and a cytosol fraction [12]. The uncoating machinery comprises two components: a DnaJ protein that binds specifically to clathrin (auxilins 1 and 2 in mammals, also known as, respectively, neuronal auxilin and its ubiquitous homolog Cyclin‐G‐associated kinase (GAK) [13, 14, 15]; Swa2 in *S. cerevisiae* [16]; AUXILIN‐LIKE1/2 in *Arabidopsis thaliana* [17]), and an ATPase from the Heat shock cognate 71 kDa (Hsc70) protein family [18, 19]. Structural studies with mammalian proteins have shown that auxilin interacts with the terminal domain of the clathrin heavy chain, and recruits Hsc70, which then binds to a QLMLT motif located in the unstructured C‐terminal domain under the triskelion vertex [20, 21]. Then, upon ATP hydrolysis, the complex thus formed is disassembled. Two mechanistic models have been proposed for this disassembly. The “steric wedge” model is based on the structure of auxilin‐bound clathrin cages obtained with electron cryomicroscopy [22]: auxilin binds to clathrin at the ankle crossing location, which is an interaction hub between 3 and 4 clathrin triskelia. This specific localisation allows auxilin to recruit Hsc70 to the tripod region that is known to be involved in coat stability [22]. Auxilin binding induces a conformation change within the clathrin triskelia, causing large‐scale strain on the lattice, and exposing the C‐terminal segment of clathrin heavy chains to Hsc70 to stimulate its ATPase [21, 22]. These findings are corroborated by both in vitro light‐scattering assays as well as single‐particle fluorescence microscopy of clathrin cages: both highlight a lag phase between auxilin/Hsc70 initial recruitment to clathrin cages and uncoating, supporting destabilisation through accumulation of strain and distortions within the coat [23, 24]. In the other model, the clathrin structure is destabilised by entropic pulling and collision pressure forces [25]. This model dictates that ATP‐bound Hsc70 recruitment to clathrin coats by auxilin would bring it in close proximity to the “wall” formed by assembled clathrin legs, which would induce repeated cycles of entropic pulling and pushing between the clathrin wall and Hsc70, thereby imparting strain on the clathrin assembly, and eventually driving the triskelia apart. Taken together, these two models suggest that auxilin recruits Hsc70 to optimal positions on the clathrin lattice for its disassembly. The auxilin/Hsc70 machinery then induces conformational changes and generates entropic pulling forces that both accumulate strain, until triskelia are released from the coat [26].

The activity of the uncoating machinery upon vesicle scission is well established: in mammalian cells, live fluorescence microscopy shows a burst of auxilin recruitment at the end of the endocytic process [27]. Moreover, DnaJ deletion experiments in budding yeast were shown to be associated with a strong defect in clathrin‐coated vesicle (CCV) uncoating in the cytosol [16]. However, early efforts to characterise clathrin assembly at endocytic sites highlighted a paradox with this late recruitment: clathrin polymers involve intricate interactions between the legs of multiple triskelia, making their assemblies highly stable [28]. Such stable clathrin assembly appears contradictory to the dynamicity of endocytic vesicle formation, and the large‐scale changes in clathrin lattice organisation that were suggested by early EM studies. One of the most striking examples of this lattice reorganisation was presented in a study by Larkin et al. [29]. They arrested clathrin‐coated pit (CCP) formation by potassium depletion and used electron microscopy to assess the changes in clathrin lattice architecture after relief of this potassium depletion‐induced CCP arrest. They observed that, as the time after CCP maturation recovery got longer, flat clathrin lattices grew, and this expansion was followed by a rise in the prevalence of pentagon‐containing, curved clathrin structures [29]. Such a large‐scale reorganisation necessarily entails removal and reassembly of the clathrin triskelia in the lattice. However, these results present an important caveat: these early studies were based on the electron microscopy of fixed cells, rather than following the assembly dynamics of single endocytic sites. It could thus be argued that these various clathrin assemblies could be an artifact of the sample preparation process for EM imaging, or could correspond to distinct biological structures, without any temporal link between them. This latter argument is further supported by the coexistence of dynamic CCPs and stable clathrin plaques in many cell types, including fibroblasts [30]. These plaques were shown not to be endocytic sites, but related to cell adhesion and structural functions [30, 31, 32, 33, 34]. To reconcile the high stability of clathrin structures with the apparent dynamicity of endocytic vesicle formation, we need to understand the dynamic properties of clathrin assemblies in living cells.

### 2.3 Clathrin Dynamics During Endocytic Site Formation

Progress in live‐cell microscopy techniques has enabled the visualisation of fluorescently labelled proteins and the study of their behaviour under endogenous physiological conditions. Fluorescence Recovery After Photobleaching (FRAP) experiments have revealed that, in mammalian cells, clathrin molecules turn over at the endocytic sites, with fluorescence recovery time constants of 18.9 ± 1.0, 10.6 ± 1.9 and 15.9 ± 1.0 s for human light chain A (LCa), light chain B (LCb) and heavy chain, respectively [35]. These experiments have also highlighted a mobile fraction of about 60% of the clathrin molecules assembled at single endocytic sites [35, 36]. Both clathrin heavy and light chains were shown to exchange at similar rates, which suggests that the units turning over are mostly the clathrin triskelia, and not heavy and/or light chains independently [35]. The turnover of clathrin was observed both in early CCPs prior to the recruitment of dynamin and late CCPs containing both clathrin and dynamin [36]. The exchange of clathrin triskelia appears to be a conserved behaviour as clathrin was also shown to be highly dynamic in fission yeast using single‐molecule speckle (SiMS) microscopy, with a single‐molecule average residence time of only ~1.5 s [37]. Interestingly, clathrin's dynamic behaviour was found to be endergonic: in ATP‐depleted cells, both clathrin turnover and endocytosis were arrested [11], which strongly suggests that the uncoating machinery is not only at play during the final CCV disassembly step, but also during the early stages of coat formation.

In addition to clathrin, several other endocytic proteins have been shown to undergo rapid turnover during endocytic events: a scaffold protein Eps15 in mammals, and its yeast homolog Ede1 [38, 39, 40], clathrin adaptor protein AP‐2 [41], and yeast WASP homolog Las17 [39]. However, the requirement of an external mechanism to regulate the assembly of a coat protein in an ATP‐dependent fashion has only been observed with clathrin.

The conservation of this controlled, endergonic turnover suggests that it may have a crucial function in the regulation of endocytic progression; however, the exact nature of such function is yet to be fully deciphered.

## 3 Is Clathrin Turn‐Over a Compromise Between Uncoating Efficiency and Specificity?

One possible explanation for the recruitment of the uncoating machinery to growing endocytic sites is that it is an evolutionary compromise between the requirement for a highly effective and fast‐acting uncoating mechanism of CCVs, and the specificity of this mechanism to assembled clathrin structures. Indeed, Hsc70 alone cannot disassemble clathrin cages, and requires the clathrin‐specific DnaJ for its uncoating activity [42]. Additionally, structural studies have shown that the DnaJ and Hsc70 proteins bind to opposite ends of the clathrin heavy chain, thus restricting the uncoating complex formation to assembled clathrin structures [20, 21, 43]. The use of tracer molecules has revealed that the fusion of endocytic vesicles with early endosomes occurs very rapidly, typically within ~1–2 min after uptake [44]. Moreover, an in vitro study by Böcking et al. evaluated an [Hsc70]‐dependent rate constant k ≈ 0.105 μM‐1 s‐1 for the CCV disassembly phase. Since the cytosolic concentration of Hsc70 typically ranges from 20 to 30 μM, this implies a clathrin cage disassembly occurring within less than half a second post Hsc70 binding [23]. Ensuring the reliability of the uncoating process might have evolved at the cost of its specificity: the uncoating machinery could be recruited weakly to all assembled clathrin structures, and not only CCVs. In this context, the early‐stage clathrin turnover would not bear any direct biological function, but would simply denote a leakage in the Hsc70/DnaJ uncoating activity. However, a growing body of evidence does indicate that preventing clathrin exchange at endocytic sites has detrimental effects on CCP formation, which rather suggests a direct function for clathrin dynamics in the regulation of endocytic site formation [11, 45, 46].

## 4 Structural Functions for the Early‐Stage Clathrin Turnover During CCP Formation

### 4.1 Clathrin Dynamics Regulate Membrane Deformation

ATP depletion both stops clathrin exchange at endocytic sites and arrests CCP maturation, inhibiting the vesicle budding step [11]. More recent studies have confirmed the crucial role played by the uncoating machinery in coat maturation and vesicle formation, in both epithelial and neuronal cells [45, 46]. This function in membrane deformation could be directly linked to clathrin's structural and assembly properties: clathrin is known to be one of the first proteins to be recruited to the endocytic site [47, 48], so its initial assembly could occur as a flat structure. In fact, clathrin is known to first assemble on a flat membrane in budding yeast [49, 50]. Studies in mammalian cells have shown that clathrin assembly correlates with a progressive increase in membrane curvature over time [36, 51]. Since clathrin can assemble into flat lattices that are fully hexagonal, subsequent accommodation for membrane deformation entails the introduction of pentagonal openings in the structure, which can only be achieved by removing and/or reassembling a number of triskelia within the structure [52]. The clathrin turnover observed in cells could therefore underlie this transition from hexagonal to pentagon‐containing coats. This transition is possibly illustrated by the recurring observation of heptagonal openings juxtaposed with pentagons in clathrin arrays with low levels of curvature in unroofed fibroblasts [53]. Since the presence of heptagons in highly curved coats and fully formed clathrin cages is a fairly rare occurrence [53, 54], these heptagons could be a property of an intermediary state of the nascent CCP.

However, whether clathrin dynamics accommodate membrane deformation, or actually drive it, remains unclear. Although ATP depletion was shown to arrest CCP maturation in mammalian cells, inhibition of Hsc70/auxilin‐mediated clathrin turnover at CCPs in cell‐free reconstitution systems did not appear to hamper membrane deformation [55]. Furthermore, investigating the impact of clathrin deletion on the endocytic site maturation process in budding yeast showed that endocytic vesicle formation still occurred [56], casting even more doubt on clathrin's direct role in membrane deformation. Since clathrin is well‐conserved across many organisms, one could expect its assembly properties and its function at endocytic sites to be fairly conserved as well, which is further supported by the presence of the clathrin uncoating machinery across many phyla. However, the hypothesis that conservation of clathrin indicates a similar function in various organisms, might represent an overly restrictive and simplified view of the correlation between the conservation of an endocytic protein structure, and that of the endocytic process as a whole. While endocytic site formation in mammals and yeast follows similar steps, a closer look reveals fundamental differences. Notably, while the onset of membrane deformation in mammalian endocytosis is still debated and appears to be variable depending on the cell properties and culture conditions [51, 57, 58, 59, 60, 61], membrane deformation in yeast cells is highly stereotypical, and consistently occurs within the last ~10 s of the whole endocytic process, which itself lasts 1–2 min [50, 62, 63]. Membrane deformation in yeast was also shown to largely depend on actin polymerisation [62]. This suggests that in yeast, clathrin, which is recruited long before the actin machinery to the endocytic site, necessarily assembles on a flat membrane, and later undergoes a clear and rapid flat‐to‐curved transition. However, the structure of the clathrin coat has never been observed at endocytic sites in yeast cells, because of the challenges in visualizing the small yeast endocytic coat. Therefore, the dynamic behaviour of clathrin might simply reflect its assembly into a loose structure rather than an ordered lattice. Data on clathrin dynamics in yeast is much sparser than in mammalian cells. Newpher et al. studied the fluorescence loss after photobleaching (FLIP) of membrane‐associated GFP‐clathrin signal in budding yeast, and showed a dynamic behaviour [48]. More recently, Lacy et al. have estimated a fast clathrin turnover at endocytic sites in fission yeast using SiMS [37]. Further studies in the yeast models are needed: although the effect of the uncoating machinery on CCVs is well established, its recruitment during the early stage of endocytic site formation has never been observed in yeasts [64].

The hypothesis of a loose early clathrin coat assembly is further supported by studies of endocytic coat structure in mammalian cells, which showed that the early‐stage clathrin coats are not fully hexagonal. A study by Sochacki et al. investigating clathrin coat assembly in cells using platinum replica EM highlighted a significant number of “imperfect” clathrin lattices, which contained breaks and missing triskelia. They suggested that these apparent breaks in the flat lattices could correspond to pentagons being pre‐loaded into the structure, in order to facilitate the subsequent curvature generation [65]. Vacancies in clathrin lattices were also shown to lower the energy cost of curvature generation in computer simulations [66]. One could therefore imagine a scenario whereby the uncoating machinery preferentially removes triskelia tightly bound to one another, thereby maintaining a partially loose structure that can accommodate changes in curvature more easily. In this situation, clathrin turnover would not drive membrane deformation, but rather allow it to happen. This would reconcile the apparently distinct roles clathrin seems to play in membrane deformation in both yeast and mammals: because membrane deformation in yeast is primarily driven by actin polymerisation, clathrin deletion does not hinder vesicle budding [56]. In mammalian endocytosis however, the actin network is thought to only play an essential role when membrane tension is high [67]. When membrane tension is moderate, clathrin dynamics could maintain a loosely assembled clathrin coat. This loose coat would facilitate the onset of membrane deformation, at which point the coat would adopt a curved, pentagon‐containing configuration that could, in turn, stabilise the nascent curvature of the membrane. Therefore, in absence of clathrin, the unstable membrane deformation would lead to failed vesicle budding. This hypothesis is supported by the striking observation that, upon generation of stable curvature in the plasma membrane via micropatterned substrates, endocytosis can proceed in mammalian cells even upon clathrin knockdown [68].

It is also possible that clathrin has evolved to play distinct functions in yeast and mammalian endocytosis. Although similar structures between homologous proteins can usually be paired with similar functions, exceptions to this rule do exist. However, an overwhelming majority of homologous endocytic proteins share very similar functions in both mammals and yeast [69], such as Eps15 and its yeast homolog Ede1 [38, 40], Fcho1/2 and yeast Syp1 [70, 71], all of which are involved in endocytic site nucleation; Hip1R and its yeast homolog Sla2, which were both shown to couple the endocytic coat to the plasma membrane and the actin network [72, 73]; N‐WASP and yeast Las17, both serving as nucleation promoting factors (NPF) in actin network polymerisation [12, 74]. It thus seems likely that clathrin in both yeast and mammals would share a similar function as well. Instead of playing a direct role in membrane deformation, the Hsc70/DnaJ‐controlled clathrin assembly could be involved in the regulation of endocytic site maturation, which, if faulty, could lead to failed vesicle budding in mammals and not in yeast, simply because of the different nature of the force to be counteracted for it to occur.

### 4.2 Coat Structure Proofreading

CCP lifetimes across species are highly variable, ranging from ~30 to ~40 s in *Arabidopsis thaliana* [75]; from ~1–3 min in *S. cerevisiae* [49]; and from ~30s to ~300 s in mammalian cells [76]. The population of short‐lived pits in mammalian endocytosis are thought to be abortive endocytic events, that is, events where the nascent coat is rapidly disassembled without giving rise to the formation of a vesicle. It was shown that CCPs are nucleated stochastically and need to be stabilised lest they be rapidly disassembled [77]. This stabilisation mechanism was proposed to be linked to cargo capture [55, 77].

Interestingly, expressing mutated AP‐2 complex subunit α‐adaptin lacking its appendage domain was shown to perturb curvature generation; instead, clathrin assembled as flat lattices turning into abortive endocytic events [78]. The α subunit of the AP‐2 complex binds to many endocytic accessory proteins, including auxilin [79]. Other α subunit binders include amphiphysin, Eps15, epsin, and AP180/CALM [79], which are all involved in membrane deformation: Eps15 is a largely disordered protein that interacts with another early‐arriving endocytic protein, FCHo1/2 [71]. Both FCHo1/2 and amphiphysin contain BAR domains, which are crescent‐shaped domains inducing membrane curvature [71]. Both FCHo1/2 and amphiphysin were shown to play a direct role in membrane remodelling during endocytosis [71, 80]. Epsin and AP180/CALM were also found to be drivers of membrane curvature via the amphipathic helices located in their ENTH and ANTH domains, respectively, which were shown to wedge into the membrane, thereby inducing its deformation [81, 82, 83, 84, 85]. Auxilin and these various curvature‐generating proteins interacting with the same subunit of the AP‐2 complex could underlie a mechanism by which auxilin‐mediated clathrin uncoating and endocytic protein recruitment regulate each other and, consequently, membrane deformation. This suggests an intricate interplay between the regulation of clathrin assembly, accessory protein recruitment and membrane bending, going beyond each protein's structural and mechanical properties.

## 5 Regulatory Functions for the Early‐Stage Clathrin Turnover

### 5.1 Clathrin Dynamics and Cargo Recruitment

#### 5.1.1 Interplay Between Clathrin Turn‐Over and Cargo Capture

The endocytic site is characterised by the complex balance of structured and highly flexible components, all coming into play at different times and interacting with one another with varying strengths. The Hsc70/DnaJ‐driven clathrin exchange could be a mechanism to prevent an overly stable clathrin assembly; therefore, maintaining a partial dynamicity of the endocytic site as a whole. This dynamicity could help promote and/or regulate protein diffusion and recruitment to the site, particularly cargo molecules. In fact, Chen et al. showed, in a cell‐free reconstitution system consisting of a cell‐derived plasma membrane incubated with a cytosolic fraction, that preventing clathrin exchange at CCPs by mutations in auxilin or Hsc70 completely abolished transferrin receptor TfR clustering at CCPs [55]. Cargo capture is known to play a role in endocytic site maturation: overexpression of the human TfR was shown to reduce the proportion of early abortive events [86], while the absence of ubiquitination of μ‐opioid neuropeptide receptors (which is the endocytic signal they rely on for their internalisation) was found to hamper endocytic maturation [87]. Additionally, inhibition of the secretory pathway in yeast cells increased significantly the lifetime of endocytic events [88], suggesting a conserved role for cargo recruitment in endocytic site maturation.

An interplay between clathrin lattice remodelling and cargo selection was also highlighted at flat clathrin lattices. Although these long‐lived structures were found to be involved in several cell processes such as cell growth, adhesion and migration [33, 89], several studies have highlighted internalisation events occurring within or in the immediate vicinity of clathrin plaques [53, 60, 90, 91, 92]. Moreover, clathrin triskelia forming these lattices were found to turn over in an ATP‐dependent fashion [90]. A study conducted by Leyton‐Puig et al., combining super‐resolution microscopy and live‐cell TIRF microscopy, showed that activation of specific receptors, such as the LPA receptor 1, correlated with actin network‐mediated plaque disassembly and CCV formation, suggesting that the flat clathrin plaques could serve as cargo‐selecting hubs for CCV formation. These findings are corroborated by the frequent observations in unroofed cells imaged by EM, of heptagonal and pentagonal openings at the edges of flat clathrin lattices [53]. These non‐hexagonal shapes were proposed to represent intermediate states of the curving clathrin lattice, which emphasizes the tight interplay between clathrin dynamics, cargo recruitment and membrane deformation. However, the role played by clathrin dynamics in cargo recruitment possibly results from an indirect mechanism. Clathrin itself does not bear any known cargo binding regions. However, most clathrin adaptors interact directly with cargo molecules via various endocytic signals. For instance, in mammals the transferrin receptor TfR is recognised by the AP‐2 complex via a YxxØ motif (with x representing any amino acid, and Ø representing any bulky hydrophobic residue) [93, 94, 95]. And much like clathrin, several adaptor proteins were found to be dynamic at endocytic sites: mammalian AP‐2 was shown to exchange rapidly in live‐cell FRAP experiments [41]; and photobleaching yeast clathrin adaptors Yap1801/2 and AP‐2 subunit Apl1, results in a partial signal recovery [39, 96]. This adaptor protein exchange could be a mechanism for cargo capture: one could imagine a scenario whereby “free” adaptors turn over, whereas those bound to cargo become more stable. This differential exchange between cargo‐bound and cargo‐free adaptor proteins could be mediated by clathrin turnover. While clathrin turnover was shown to occur throughout endocytic site maturation in mammalian cells [36], it remains unclear whether adaptor protein dynamics change over time. Nevertheless, AP‐2 binding to YXXφ‐containing cargo was found to change its conformation, impacting both clathrin recruitment and CCP maturation [97]. This highlights a direct interplay between clathrin‐to‐adaptor binding and cargo‐to‐adaptor binding, which could be tied with clathrin and/or adaptor dynamics. However, clathrin and adaptor dynamics appear largely independent of one another. While ATP depletion completely arrests clathrin exchange, it was shown not to affect AP‐2 dynamics [41]. Whether clathrin adaptors are still able to capture cargo molecules upon ATP depletion remains unclear. The apparent independency between clathrin and adaptor dynamics could hint at a complex interplay between clathrin exchange and cargo recruitment, that might rely on other yet‐to‐be‐identified players.

#### 5.1.2 Regulation of Protein Assembly: Optimal Clathrin: Adaptor Ratio

Clathrin dynamics could also be involved in the regulation of adaptor assembly at the endocytic site. The clathrin:adaptor ratio is a crucial aspect in the regulation of endocytic site formation, as multiple studies have shown that it can directly impact coat stiffness and, therefore, the onset of membrane deformation [51, 98]. A controlled clathrin turnover could thus serve as a mechanism modulating adaptor assembly and coat stiffening, thereby ensuring sufficient time for cargo capture, which, as stated before, could also be a contributing factor in the stiffening of the coat, highlighting the complex interplay between these endocytic components.

#### 5.1.3 Regulation of Protein Assembly: Limiting Cargo Crowding

The significance of the interplay between clathrin dynamics and cargo selection in the regulation of CCP formation is emphasized by the additional role cargo amounts play in membrane deformation: many cargo molecules are transmembrane proteins exhibiting a strong asymmetry from one membrane leaflet to the other. Quite notably, numerous receptor proteins such as low‐density lipoprotein (LDL) receptors [99], integrins [100] or immune receptors exhibit large ectodomains. These bulky extracellular domains exert a significant steric pressure on the side of the outer membrane leaflet, which would oppose membrane deformation, hampering CCV budding [101]. In fact, the steric pressure exerted by cargo molecules, and its direct impact on membrane deformation, was shown to play a role in the regulation of COPII‐coated vesicle trafficking [102]. Therefore, regulating the number of cargo molecules per pit may also be crucial to facilitate membrane bending. This suggests a restricted window for the number of cargo molecules at endocytic sites; the control of clathrin dynamics by the Hsc70/DnaJ machinery could play a role in the regulation of cargo recruitment to fit in that optimal window.

### 5.2 Clathrin Assembly and Lipid Composition

The lipid composition of the plasma membrane plays a key role in both endocytic site initiation and maturation [71, 103], and lipids are potent drivers of membrane deformability [104]. Notably, Phosphatidylinositol‐4,5‐bisphosphate (PIP2) lipids, which are found in high concentrations on the inner leaflet at endocytic sites, are essential actors of CCP formation, as they are the main lipid species many endocytic proteins bind to, such as endocytic site nucleators FCHo1/2 [71], the AP2 complex [105], as well as ENTH‐ and ANTH‐domain‐containing proteins (for instance, respectively, epsins [82] and AP180/Yap180s [106]). Interestingly, the AP‐2 complex was found to recruit Type I phosphatidylinositol 4‐phosphate 5‐kinase gamma (PIPKIɣ) to endocytic sites via its β2 appendage, which also binds to clathrin, with a higher affinity for the latter [107]. Therefore, it is possible that selectively removing clathrin triskelia might help promote the recruitment of PIPKIɣ to endocytic sites and, with it, the enrichment of PIP2. Binding to PIP2 was also shown to anchor AP‐2 to the endocytic sites, and trigger a conformation change into an open state that recruits clathrin. This suggests that selectively removing clathrin triskelia might actually promote further clathrin assembly at endocytic sites.

Polyunsaturated phospholipids were shown to adapt their conformation to membrane curvature, decreasing the energy cost limiting dynamin's and endophilin's ability to deform membranes [108]. Therefore, clathrin dynamics could indirectly promote membrane deformation via PIP2‐enrichment, which further highlights the complexity of protein assembly regulation at the endocytic site.

### 5.3 Clathrin Assembly and Spatial Organisation of Endocytic Proteins

Controlling the assembly of endocytic proteins does not only mean controlling their amount, but also their organisation, which can involve highly stereotypical and well‐conserved spatial patterns. For instance, while clathrin can generally be found at the centre of the site, many proteins, including those associated with actin assembly, primarily assemble at the rim of the clathrin coat in both yeast and mammalian endocytosis [73, 109, 110]. This intricate architecture illustrates the central role played by clathrin: as a stiff scaffolding protein, clathrin interacts with many other endocytic players and helps drive their localisation pattern at the site. This function was illustrated in two complementary studies, which showed that interaction between the clathrin light chain and homologs Hip1R and Sla2 is involved in timing and directing actin polymerisation at the rim of the clathrin lattice [67, 111]. Therefore, controlling clathrin assembly by the uncoating machinery could be crucial, as other proteins might depend on the size and shape of the clathrin coat for their correct recruitment. Moreover, the specific recruitment pattern of many proteins involved in vesicle budding at the rim of the clathrin lattice [110, 112] hints at a role for clathrin in regulating the overall size of the CCP. The strongest evidence of clathrin playing a crucial role in regulating the size of endocytic sites can be found in budding yeast: The size of endocytic vesicles is tightly controlled in wild type yeast cells. In clathrin deletion cells the endocytic vesicles still form, although in reduced numbers, and they have normal average size, but the size variation is greatly increased [56]. Hints of the conservation of this function were also found in other organisms: in *Drosophila*, mutations in adaptor protein AP180 homolog LAP (Like AP180) impaired clathrin localisation in presynaptic boutons, resulting in a reduced number and increased size variability of vesicles [113]. Mammalian AP‐180 was also shown to regulate the size of clathrin baskets [114]. Moreover, in mammalian endocytosis, clathrin coat assembly time was shown to scale with the size of cargoes to be internalised; for instance, large objects like virus particles were associated with a significantly longer clathrin assembly time and, with it, a delayed internalisation, demonstrating that clathrin assembly can be modulated to adapt the size of the cargo to be internalised [77]. This latter finding emphasizes the importance of a dynamic control of clathrin assembly to ensure the responsiveness and adaptability of the endocytic process to various cues such as cargo recruitment time and cargo size.

## 6 Perspectives

The mechanisms governing clathrin assembly and its implications in the regulation of the CCP formation have been longstanding questions in the field of endocytosis. Many of clathrin's structural properties involved in its self‐assembly as well as its interaction with numerous endocytic partners have been elucidated in detail. However, how these properties come into play dynamically to control the assembly of the endocytic machinery is still mostly uncharted territory. Early hypotheses proposed that clathrin exchange served a purely structural role, sculpting the endocytic pit and driving membrane deformation. However, live‐cell imaging and genetic manipulation techniques have revealed clear connections between clathrin dynamics and the maturation of the endocytic process. Despite this progress, where the clathrin turnover falls within the regulatory network governing endocytosis remains unresolved. A major obstacle to clarifying its function is that experiments performed under different conditions (i.e., in vitro vs. in cells) often yield divergent outcomes. In this review, we have examined the various roles and interactions attributed to clathrin dynamics, many of which appear to act at distinct time points during endocytosis. Taken together, these observations suggest that clathrin turnover may have multiple downstream effects on different components of the endocytic machinery, each contributing in specific ways to maturation. Rather than a simple one‐cause–one‐effect relationship, clathrin dynamics may instead operate within a complex, interconnected matrix of regulatory cues (**Figure** **1**).

Another major hindrance to a better understanding of the precise function of clathrin dynamics is the multiplexity of the protein: clathrin is not only involved in endocytosis, but also in other membrane trafficking steps such as trans‐Golgi network trafficking. The same is true for its uncoating machinery, which was also found to disassemble coats from Golgi‐derived CCVs [15]. Therefore, dissecting their function at endocytic sites specifically by manipulating Hsc70's and/or DnaJ's interaction with clathrin for instance, proves to be particularly challenging. The partial uncoating observed during endocytic site formation, as opposed to the complete coat disassembly after budding, could suggest a weaker activity and/or affinity of the Hsc70/DnaJ machinery for clathrin structures exhibiting less curvature. Generating Hsc70 and/or DnaJ mutants with a weaker affinity for clathrin might therefore abolish the early‐phase turnover without hampering the final uncoating step, which could be a strategy to investigate the specific function of this early‐stage clathrin exchange. Another question that remains unanswered is that of a potential variability of clathrin dynamics between endocytic events, which was also observed in distinct cell types [115], or throughout the endocytic site maturation lifetime. FRAP experiments have yielded similar dynamic properties for clathrin at both early and late CCPs, however it is worth noting that these results were obtained assuming steady state conditions, because FRAP experiments cannot easily differentiate protein recruitment from protein exchange. SMIT techniques could help bridge this gap—however these techniques do have their own limitations [37].

Variability in clathrin dynamics is likely, because endocytic events themselves are variable and the endocytic machinery undergoes numerous changes during single events. Notably, one of the isoforms of human clathrin light chain, CLCb, was shown to directly affect endocytic site maturation depending on its phosphorylation state: unphosphorylated CLCb increases the prevalence of early endocytic event termination [116]. Interestingly, these abortive events were also shown to recruit DnaJ protein GAK; this might point to an interplay between the phosphorylation state of clathrin and its interaction with its uncoating machinery, which could suggest a direct effect of clathrin phosphorylation on clathrin turnover. The study by Maib et al. also showed that the unphosphorylated‐CLCb‐associated abortive events failed to cluster cargo, which strongly suggests that clathrin dynamics might not have just one role but could contribute to a combination of various regulative processes to ensure successful CCP formation. It appears clear that understanding the exact function of clathrin turnover will only be possible by carefully considering a broad and dynamic picture of the full endocytic network throughout CCP formation. Albeit challenging, this careful and systematic dissection of protein dynamics and their integration into the endocytic site regulation network might help shed light not only on the endocytic process itself, but also on the fundamental principles governing dynamic multi‐protein assemblies.

## Funding

This work was supported by Swiss National Science Foundation, 310030_212288, 310030_200793, CRSII5_189996; European Research Council, 951324‐R2‐TENSION.

## Conflicts of Interest

The authors declare no conflicts of interest.

## Data Availability

Data sharing not applicable to this article as no datasets were generated or analysed during the current study.
